# Cerebrospinal Fluid MicroRNA Changes in Cognitively Normal Veterans With a History of Deployment-Associated Mild Traumatic Brain Injury

**DOI:** 10.3389/fnins.2021.720778

**Published:** 2021-09-09

**Authors:** Theresa A. Lusardi, Ursula S. Sandau, Nikita A. Sakhanenko, Sarah Catherine B. Baker, Jack T. Wiedrick, Jodi A. Lapidus, Murray A. Raskind, Ge Li, Elaine R. Peskind, David J. Galas, Joseph F. Quinn, Julie A. Saugstad

**Affiliations:** ^1^Knight Cancer Institute, Cancer Early Detection Advanced Research Center, Oregon Health & Science University, Portland, OR, United States; ^2^Department of Anesthesiology & Perioperative Medicine, Oregon Health & Science University, Portland, OR, United States; ^3^Pacific Northwest Research Institute, Seattle, WA, United States; ^4^Biostatistics & Design Program, Oregon Health & Science University, Portland, OR, United States; ^5^Northwest Mental Illness, Research, Education, and Clinical Center, VA Puget Sound Health Care System, Seattle, WA, United States; ^6^Department of Psychiatry and Behavioral Sciences, University of Washington School of Medicine, Seattle, WA, United States; ^7^Geriatric Research Education and Clinical Center, VA Puget Sound Health Care System, Seattle, WA, United States; ^8^Department of Neurology, Oregon Health & Science University, Portland, OR, United States; ^9^Parkinson Center and Movement Disorders Program, School of Medicine, Oregon Health & Science University, Portland, OR, United States; ^10^Portland VAMC Parkinson’s Disease Research, Education, and Clinical Center, Portland, OR, United States

**Keywords:** veterans, deployment, mild traumatic brain injury, cerebrospinal fluid, microRNA, Alzheimer’s disease

## Abstract

A history of traumatic brain injury (TBI) increases the odds of developing Alzheimer’s disease (AD). The long latent period between injury and dementia makes it difficult to study molecular changes initiated by TBI that may increase the risk of developing AD. MicroRNA (miRNA) levels are altered in TBI at acute times post-injury (<4 weeks), and in AD. We hypothesized that miRNA levels in cerebrospinal fluid (CSF) following TBI in veterans may be indicative of increased risk for developing AD. Our population of interest is cognitively normal veterans with a history of one or more mild TBI (mTBI) at a chronic time following TBI. We measured miRNA levels in CSF from three groups of participants: (1) community controls with no lifetime history of TBI (ComC); (2) deployed Iraq/Afghanistan veterans with no lifetime history of TBI (DepC), and (3) deployed Iraq/Afghanistan veterans with a history of repetitive blast mTBI (DepTBI). CSF samples were collected at the baseline visit in a longitudinal, multimodal assessment of Gulf War veterans, and represent a heterogenous group of male veterans and community controls. The average time since the last blast mTBI experienced was 4.7 ± 2.2 years [1.5 – 11.5]. Statistical analysis of TaqMan^TM^ miRNA array data revealed 18 miRNAs with significant differential expression in the group comparisons: 10 between DepTBI and ComC, 7 between DepC and ComC, and 8 between DepTBI and DepC. We also identified 8 miRNAs with significant differential detection in the group comparisons: 5 in DepTBI vs. ComC, 3 in DepC vs. ComC, and 2 in DepTBI vs. DepC. When we applied our previously developed multivariable dependence analysis, we found 13 miRNAs (6 of which are altered in levels or detection) that show dependencies with participant phenotypes, e.g., ApoE. Target prediction and pathway analysis with miRNAs differentially expressed in DepTBI vs. either DepC or ComC identified canonical pathways highly relevant to TBI including senescence and ephrin receptor signaling, respectively. This study shows that both TBI and deployment result in persistent changes in CSF miRNA levels that are relevant to known miRNA-mediated AD pathology, and which may reflect early events in AD.

## Introduction

Traumatic brain injury (TBI) has been associated with cognitive impairment (McInnes et al., 2017). Several epidemiologic studies have also reported an association between a history of closed head injury and Alzheimer’s disease (AD) or dementia (Plassman and Grafman, 2015). Repetitive, mild TBI (mTBI), resulting from either blunt force and/or blast wave brain trauma, are particularly strong predictors of AD and related disorders (ADRD) (Guskiewicz et al., 2005; DeKosky et al., 2010). Retrospective cohort studies in older veterans demonstrated that TBI in earlier life was associated with a 60% increase in the risk of developing dementia (Barnes et al., 2014, 2018). Since the year 2000, the Department of Defense has recorded over 300,000 TBIs in service members deployed to Iraq and/or Afghanistan, and it is likely that mTBIs in particular have been under-reported (Sibener et al., 2014). The United States Department of Veterans Affairs recently published a review of the evidence regarding the link between TBI and dementia: www.hsrd.research.va.gov/publications/esp/tbi-dementia-brief.pdf. This review reported that while few studies have focused on evaluating the association between TBI and dementia in military and veteran populations, those that did (Barnes et al., 2014, 2018) were consistent with a systematic review of worldwide general community studies that suggest TBI is associated with increased risk of dementia (Li et al., 2017). Interestingly, the Li study also demonstrated a dose-response relationship between number of TBIs and dementia diagnosis. Consequently, as the population of veterans ages, an increased incidence of ADRD will present a large burden on families, caregivers, and the Veterans Affairs health care system.

The precise mechanisms triggered by TBI that may lead to cognitive impairment are not clear. In this study, we examined miRNAs as a link between TBI and AD in war veterans. MiRNAs are short, non-coding RNAs that regulate protein translation via suppression or degradation of messenger RNA (mRNA) transcripts (Bushati and Cohen, 2007; Filipowicz et al., 2008; Shyu et al., 2008). MiRNAs contribute to normal developmental changes in the brain, and to the initiation and/or progression of neurological diseases in neurons and glia (O’Carroll and Schaefer, 2013). Furthermore, changes in miRNA levels have been linked to neurodegeneration across many diseases (Juzwik et al., 2019), including AD (Wang et al., 2019). Circulating cell-free miRNAs also serve as sensitive markers for changes in neurological diseases, including AD (Batistela et al., 2017) and TBI (Atif and Hicks, 2019). Here we focused on analyzing cerebrospinal fluid (CSF) given that it bathes and reflects the state of the brain, and that CSF miRNAs have been reported as biomarkers for AD (Lusardi et al., 2017; Denk and Jahn, 2018; McKeever et al., 2018; Wiedrick et al., 2019; Sandau et al., 2020b).

The time interval between brain injury and the potential onset of ADRD symptoms is likely to be years or decades long. During this interval, confounding influences may complicate the development of predictive prognostic tools. Thus, to effectively analyze TBI data we need to be able to identify diverse multivariable dependencies. We recently introduced an information theory-based set of measures that allow us to quantify statistical dependencies among multiple variables. These measures make no assumption about the functional nature of the dependence, thus allowing us to detect multivariable dependencies without defining the underlying function of the dependence (Galas et al., 2014; Sakhanenko and Galas, 2015; Sakhanenko et al., 2017). While information measures do not identify which variables in the dependence are cause or effect, they are an essential tool in identifying factors that may either weaken or strengthen the ultimate utility of a biomarker panel for the early detection of ADRD. Thus, we used information measures to identify dependencies among miRNAs and AD characteristics, e.g., ApoE, that contribute to ADRD risk classification.

In this study, we examined the effect of both blast mTBI and deployment on miRNA expression in CSF. In order to understand the biological responses to TBI that may lead to AD, it is necessary to identify miRNAs that are changed at post-acute times following injury. We measured CSF miRNA expression in three groups of participants: (1) community controls with no lifetime history of deployment or TBI (ComC); (2) deployed Iraq/Afghanistan veterans with no lifetime history of TBI (DepC), and (3) deployed Iraq/Afghanistan veterans with a history of repetitive blast injury (DepTBI). Individuals in the DepTBI group experienced repeated exposures to blast mTBI, but had no signs of neurocognitive disorder at the time of CSF collection, which occurred at least 1.5 years after the most recent exposure and well past the 0 – 4 week time frame reported in prior acute TBI studies. We first analyzed CSF miRNA levels in each of these three groups, and identified CSF miRNAs that had differential expression levels or were differentially detected in each group. We then considered the contribution of the apolipoprotein E e4 (*APOE4*) gene allele, age, body mass index (BMI), smoking history, and serum proteins in combination with miRNA expression using our previously developed multivariable dependence measures, to demonstrate dependencies with participant phenotypes and identify potential confounding factors in our analysis. To understand the biological relevance of identified miRNA, we compared the miRNAs identified in this study with miRNA levels reported in published studies of humans with acute TBI responses (<4 weeks), humans with AD, and those demonstrated in model systems to have a mechanistic role in TBI or dementia. Finally, target prediction and pathway analysis was used to identify top gene candidates and signaling networks regulated by the miRNAs. Together, our results show that CSF miRNA levels reflect persistent changes associated with TBI as well as changes in AD-related signaling networks. However, ongoing longitudinal evaluations are necessary to understand whether specific changes are indicative of AD risk, or whether they reveal mechanistic insight into the initiation or progression of AD.

## Materials and Methods

### Study Participants

This study was approved by the Veterans Affairs (VA) Puget Sound Health Care System and the University of Washington Institutional Review Boards. All participants provided written informed consent in accordance with the Declaration of Helsinki prior to any study procedures (World Medical Association, 2013). One hundred and fifteen participants in this study were drawn from an ongoing longitudinal multimodal assessment study of veterans with and without repetitive mTBI that has been previously described (Petrie et al., 2014). In brief, veterans from the Iraq and Afghanistan conflicts were recruited from the local VA medical center and community population (Table 1; complete data in Supplementary Table 1). Medical and psychiatric history, including current medications, was obtained both via clinician interview and review of the medical charts. Lifetime history of TBI was evaluated by careful clinical history via semi-structured interview conducted by two expert TBI clinicians simultaneously. Behavioral health diagnostic assessments for diagnoses, including substance use disorders, were based on the Structured Clinical Interview for DSM-IV (First et al., 2002). Inclusion criteria for veterans included either no lifetime history of TBI (DepC), or at least one war-zone blast or combined blast/impact exposure (DepTBI) that resulted in acute symptoms consistent with the American Congress of Rehabilitation Medicine criteria for mTBI (Kay et al., 1993). Exclusion criteria included a reported history of moderate or severe TBI, a penetrating head wound, a seizure disorder, insulin-dependent diabetes, current or past diagnosis of primary psychotic or neurodegenerative disorder, a current or past diagnosis of bipolar disorder, or a diagnosis of active substance abuse or dependence within the past 6 months. Other exclusion criteria included contraindication to lumbar puncture, or taking medications likely to affect cognitive performance.

**TABLE 1:** Study participant characteristics.

Characteristic		ComC		DepC		DepTBI	
Number		52		18		45	
Age at CSF Collection		33.4 ± 9.3		31.8 ± 7.1		34.0 ± 10.0	
Race	White	37		14		32	
	Black/African American	1		1		2	
	Asian/Pacific Islander/Native Hawaiian	9		0		3	
	American Indian/Alaskan Native	2		0		1	
	Other/NA	3		3		6/1	
BMI***		25.1 ± 3.4		26.9 ± 4.3		28.5 ± 4.4***	
Number of mTBIs		0		0		20.5 ± 28.9 [1 – 102]	
Years Since Most Recent mTBI		N/A		N/A		4.7 ± 2.2 [1.5 – 11.5]	
Smoking Status (No/Yes/Unknown)		50/2/0		17/1/0		30/14/1	
MMSE***		29.5 ± 0.6		29.1 ± 1.1		28.5 ± 1.6***	
Abeta_42_		321.4 ± 54.2		309.5 ± 70.3		316.0 ± 55.8	
tTau		42.9 ± 17.3		35.2 ± 14.1		37.5 ± 13.7	
pTau_181_		27.8 ± 9.3		28.7 ± 8.9		28.2 ± 6.4	
Abeta_4__2_:tTau		8.2 ± 2.1		9.4 ± 1.9		9.1 ± 2.1	
pTau_181_:tTau***		0.68 ± 0.15		0.84 ± 0.10**		0.80 ± 0.14**	

***APOE4 Alleles***		**Count**	**%**	**Count**	**%**	**Count**	**%**

0		37	71.1	8	44.4	30	66.7
1		13	25.0	8	44.4	9	20.0
2		1	1.9	1	5.5	1	2.2
Samples not available for genotyping		1		1		5	

Summary characteristics for the 115 study participants. Characteristic data were analyzed by ANOVA. For characteristics with significant ANOVA, we then performed a post hoc Tukey test to assess group differences in DepC or DepTBI as compared to ComC. ANOVA testing showed a significant group effect on BMI (p < 0.001); post hoc testing showed no difference in the DepC group compared to ComC, but DepTBI BMI values were significantly higher (p < 0.001). ANOVA testing showed a significant group effect on MMSE (p < 0.001); post hoc testing revealed that MMSE in the DepC group was not different from ComC, but DepTBI MMSE values were significantly lower (p < 0.001). ANOVA testing showed a significant change in the pTau_181_:tTau ratio (p < 0.05); post hoc testing showed a significant increase in both DepC (p < 0.01) and in DepTBI (p < 0.01). Mean ± SD [Range]. Significance indicated by **p < 0.01, ***p < 0.001.

### CSF Collection and CSF AD Biomarker Measurements

CSF was obtained using a minimally invasive and well-tolerated protocol with anticipated post-lumbar puncture headache incidence < 1%, consistent with protocols utilized across AD centers (Peskind et al., 2005). CSF utilized for these studies was aliquoted and frozen on dry ice at the bedside, and stored at −80°C prior to shipment on dry ice to Oregon Health & Science University. Three CSF biomarkers reflect central pathogenic processes of AD in the brain: Abeta_42_ is a marker for Abeta metabolism and plaque formation, total tau (tTau) is a marker for the neuronal degeneration formation, and phosphorylated tau (pTau) is a marker for tau hyperphosphorylation and formation of tangles. We measured levels of CSF Abeta_42,_ tTau, and pTau_181_ using the INNO-BIA AlzBio3 assay (Innogenetics Inc, Ghent, Belgium), an immunobead-based quantitative, multiplex assay for the simultaneous quantification of these three markers in human CSF, according to the manufacturer’s instructions and as previously described (Mattsson et al., 2011). Early studies that evaluated CSF levels of Abeta_42_ alone and in combination with tTau show that the combined measure of Abeta_42_:tTau meets the requirement for discriminating AD from normal aging and other specific neurologic disorders (Hulstaert et al., 1999). Subsequent studies supported that combinations of CSF markers improves diagnostic performance for AD vs. other related neurological disorders (Blennow, 2004). In addition to increased sensitivity and specificity for the combination of Abeta_42_ and tTau over either CSF marker alone, the combination of pTau:tTau also increased the sensitivity and specificity of diagnostic performance over either CSF marker alone (Blennow, 2004). Given that the combined measures of Abeta_42_, tTau, and pTau have a high diagnostic accuracy for AD, the ratio of Abeta_42_ to tTau and the ratio of CSF pTau_181_ to tTau were used for analyses, in addition to each individual biomarker.

### RNA Isolation, Amplification, and Quantitative PCR (qPCR)

Total RNA was isolated from 500 μL CSF using the mirVana^TM^ PARIS^TM^ RNA and Native Protein Purification Kit [Thermo Fisher Scientific (TFS), AM1556], with modifications (Burgos et al., 2014), as previously reported (Lusardi et al., 2017; Wiedrick et al., 2019; Sandau et al., 2020b). MiRNAs were measured using TaqMan^TM^ Array Human MicroRNA A Cards v2.1 with *n* = 1 technical replicate probe for quantitation of 377 human miRNAs, plus probes for U6 snRNA, RNU44, and RNU48 (TFS, 4398965). Total RNA was concentrated using the RNA Clean & Concentrator^TM^-5 Kit (Zymo Research, R1013) and eluted into 9 μL of RNase/DNase-free water. 3.2 μL of concentrated RNA was reverse transcribed using the TaqMan^TM^ MicroRNA Reverse Transcription Kit (TFS, 4366596) in a 7.5 μL final reaction volume with Megaplex^TM^ Reverse Transcription Primer Pool Set v2.1 A (TFS, 4444745). 5 μL of cDNA was pre-amplified with Megaplex^TM^ PreAmp Primer Pool A v2.1 (TFS, 4444748) using TaqMan^TM^ PreAmp Master Mix (TFS, 4391128). The pre-amplification products were diluted 1:2 into a final volume of 50 μL with water, and stored at −20°C. 18 μL of diluted pre-amplification product was combined with 450 μL of TaqMan^TM^ Universal PCR Master Mix II, no UNG (TFS, 4440047) and 432 μL of water, then 100 μL was loaded per port.

For verification studies we generated a Custom TaqMan^TM^ miRNA Array Card with *n* = 3 technical replicate probes for 47 human miRNAs including our identified positive and negative controls, and U6 (TFS, 4449140) (Supplementary Table 2). Total RNA from *n* = 10 biological replicates per participant group were isolated, concentrated to 6 μL, then 4.1 μL of the concentrated RNA was reverse transcribed with a Custom miRNA RT pool (TFS, 4459652) in a total volume of 15 μL. 7.5 μL of cDNA was pre-amplified with the Custom miRNA PreAmp pool (TFS, 4459660) in a total volume of 25 μL, then diluted 1:4 with RNase-free water and stored at −20°C. 18 μL of diluted pre-amplification product was combined with 225 μL of TaqMan^TM^ Universal PCR Master Mix II, no UNG and 207 μL of water, then 100 μL of product was loaded per array card port. All reverse transcription and pre-amplification reactions were performed on a Veriti^TM^ 96-Well Thermal Cycler (TFS, 4375786) and the array cards were run on a QuantStudio 12K Flex Real-Time PCR System (TFS, 4471134), using the manufacturer’s protocol for detection of miRNAs with pre-amplification.

### MicroRNA qPCR Data Processing

**FIGURE 1:**
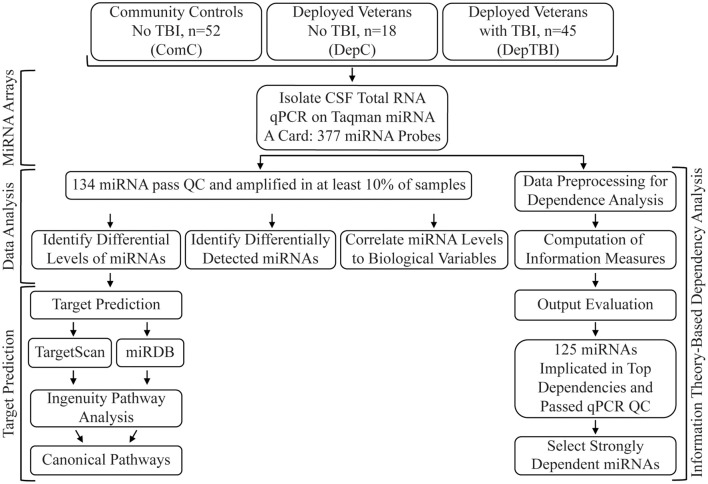
Study Workflow. The study included CSF collected from 115 veteran and community participants, with or without TBI. TaqMan array cards were used to assay 377 unique miRNAs. 134 miRNAs passed quality control (QC) metrics and were used for statistical analysis (differential expression, differential detection) and information theory (correlation to biological variables). The miRNAs with significant differential expression between each group comparison where then used for miRNA target prediction and pathway analysis. All assayed miRNA were used in the information theory dependency models, with results filtered to those that passed the initial QC step.

The complete data processing and analytic workflow is summarized in Figure 1. We updated the information for the 377 miRNA primer/probe targets on the TaqMan^TM^ Array Human MicroRNA A Card v2.1 to match miRBase, version 21 (Kozomara and Griffiths-Jones, 2014). 18 primer/probe pairs targeting sequences identified as miRNAs in previous miRbase versions were excluded from analysis because they were confirmed to be other RNA biotypes (tRNA, vault RNA) and not miRNAs, and subsequently withdrawn from miRbase (www.mirbase.org). QuantStudio^TM^ 12K Flex Software v1.3 was used for processing of the qPCR data. For each amplification we calculated the quantification cycle (Cq) value using automated baseline and threshold values determined by ExpressionSuite software v1.1 (TFS). For each miRNA the amplification score (AmpScore), which measures signal quality in the linear phase, and Cq confidence (CqConf), which measures confidence in the Cq value, were also calculated by ExpressionSuite. We evaluated each amplification based on the ExpressionSuite Cq quantification and AmpScore values. We flagged quantifications as “censored” if there was no amplification observed or the Cq value was ≥ 34. Quantifications were flagged as “excluded”, considered a technical failure, and excluded from further analysis if AmpScore < 1.0. The remaining amplifications were flagged as “good” quantifications. We considered miRNAs with either “Undetected” or Cq > 34 assignments from ExpressionSuite as below the detection threshold and censored at Cq = 34. We exported all data including the Cq, AmpScore, and CqConf out of ExpressionSuite for further analysis using R scripts. Endogenous controls (ECs) were determined according to the method of Vandesompele et al. (2002) as implemented in the NormqPCR package (Perkins et al., 2012). 15 miRNAs were considered as candidate ECs by the following criteria: (1) annotated in miRbase V.21 as a human miRNA, and (2) present in 100% of the samples with AmpScore ≥ 1, Cq < 30, and CqConf > 0.8. Candidate ECs were then ranked by their expression stability, and their contribution assessed by pairwise variation. Using the suggested threshold variation of 0.1527, we identified 3 miRNAs as ECs (denoted as hsa-miR-X-TFS Probe Number): hsa-miR-222-002276, hsa-miR-342-3p-002260, and hsa-miR-146a-000468. For normalization within each card (one study participant), we calculated ΔCq as the Cq minus the geometric mean of the ECs, for each amplification on the card. We excluded miRNAs with failed amplifications in > 20% of samples as technical error with the primer/probe. Of the remaining miRNAs, we analyzed those that were detected in at least 10% of the samples (134 miRNAs in total). For each amplification, we reported the flags, Cq, and ΔCq values in Supplementary Table 3. Flag status is summarized by experimental group in Supplementary Table 4.

### Analysis of Differentially Expressed MiRNAs

Differential expression levels of the ΔCq for 134 miRNAs were evaluated by log-rank test (R packages survival v3.1-8 and survminer, v0.4.6^1^). Fold changes and confidence intervals were calculated according to the method 2^–ΔΔ*Cq*^ (Applied Biosystems Guide to Performing Relative Quantitation of Gene Expression Using Real-Time Quantitative PCR, 4371095 Rev B). False discovery rates (Benjamini and Hochberg, 1995) were calculated, and are included in Supplementary Table 5.

### Analysis of Differentially Detected MiRNAs

To determine whether detection of a specific miRNA level differs significantly across the participant groups, we also assessed differential detection of the Cq for all 134 miRNAs. We used Fisher’s exact test, which can determine the odds of detecting significant differences in a specific miRNA across the groups. The odds ratios were calculated for the DepTBI vs. ComC, DepC vs. ComC, and DepTBI vs. DepC groups. Calculations are included in Supplementary Table 6.

### Effect of Biological Variables

For miRNAs that showed significant changes in levels or detection (Tables 2, 3), we assessed the correlation between miRNA level changes and biological variables in the participants including age at CSF collection, Mini-Mental State Examination (MMSE) scores, BMI, current tobacco use, number of TBIs, years since most recent TBI, Abeta_42_:tTau ratio, and pTau_181_:tTau ratio. We also assessed biological variables by linear regression with the miRNA expression as the outcome and the variable of interest as the predictor (uncorrected *p*-value and R2 are reported in Supplementary Table 7).

**TABLE 2:** Differential Expression Levels of CSF MiRNAs by TBI and Deployment Status.

	A. DepTBI vs. ComC		B. DepC vs. ComC		C. DepTBI vs. DepC	
miRNA	FC (CI)	*p*-value	FC (CI)	*p*-value	FC (CI)	*p*-value
502-3p	**1.01 (0.33 - 3.08)**	**0.0141**	0.56 (0.22 - 1.40)	0.6821	1.80 (0.59 - 5.50)	0.0552
362-3p	**0.80 (0.32 - 2.01)**	**0.0102**	0.59 (0.26 - 1.31)	0.5661	1.37 (0.55 - 3.42)	0.1017
191-5p	**0.76 (0.25 - 2.28)**	**0.0046**	0.88 (0.30 - 2.59)	0.9084	**0.86 (0.28 - 2.58)**	**0.0226**
197-3p	**0.66 (0.19 - 2.32)**	**0.0013**	0.71 (0.24 - 2.10)	0.1119	0.93 (0.26 - 3.25)	0.2927
30c-5p	**0.66 (0.16 - 2.69)**	**0.0239**	0.81 (0.23 - 2.84)	0.5724	0.81 (0.20 - 3.33)	0.2247
140-5p	**0.65 (0.15 - 2.88)**	**0.0038**	0.89 (0.27 - 2.93)	0.7016	0.73 (0.17 - 3.25)	0.0972
30b-5p	**0.63 (0.14 - 2.82)**	**0.0187**	0.76 (0.22 - 2.60)	0.4784	0.82 (0.18 - 3.70)	0.2218
20a-5p	**0.55 (0.13 - 2.41)**	**0.0040**	0.60 (0.16 - 2.32)	0.0787	0.91 (0.21 - 4.00)	0.6109
548a-3p	**0.50 (0.15 - 1.73)**	**0.0011**	**0.39 (0.12 - 1.30)**	**0.0149**	1.29 (0.38 - 4.44)	0.7829
20b-5p	**0.50 (0.11 - 2.19)**	**0.0007**	**0.43 (0.13 - 1.46)**	**0.0065**	1.15 (0.26 - 5.03)	0.7461
152-3p	1.28 (0.24 - 6.80)	0.1885	**2.47 (0.76 - 8.06)**	**0.0180**	**0.52 (0.10 - 2.75)**	**0.0106**
132-3p	0.88 (0.13 - 6.07)	0.9995	**2.19 (0.48 - 9.91)**	**0.0083**	**0.40 (0.06 - 2.77)**	**0.0067**
362-5p	0.68 (0.26 - 1.78)	0.3353	**1.02 (0.31 - 3.41)**	**0.0155**	**0.66 (0.25 - 1.74)**	**0.0010**
518d-3p	0.67 (0.26 - 1.76)	0.1063	**0.51 (0.22 - 1.16)**	**0.0175**	1.33 (0.51 - 3.48)	0.1295
548c-3p	0.83 (0.22 - 3.09)	0.7281	**0.34 (0.10 - 1.12)**	**0.0003**	**2.42 (0.65 - 9.01)**	**0.0011**
125a-5p	0.90 (0.22 - 3.67)	0.9223	1.30 (0.37 - 4.51)	0.0261	**0.69 (0.17 - 2.83)**	**0.0152**
130a-3p	0.79 (0.13 - 4.98)	0.8177	1.78 (0.36 - 8.77)	0.0994	**0.45 (0.07 - 2.81)**	**0.0234**
411-5p	0.66 (0.24 - 1.80)	0.2191	0.82 (0.25 - 2.74)	0.2116	**0.81 (0.30 - 2.19)**	**0.0242**

*MiRNAs with differential levels in each participant group were determined by log-rank testing of ΔCq values, and then fold changes calculated according to the −ΔΔCq method (Methods). (A). Bold font indicates miRNAs significantly altered by TBI when comparing the DepTBI vs. ComC groups. (B). Bold font indicates the miRNAs significantly altered by deployment when comparing the DepC vs. ComC groups. (C). Bold font indicates the miRNAs significantly altered by TBI when comparing the DepTBI vs. DepC groups. Results include the fold change (FC) with 95% confidence interval (CI), and p-value.*

**TABLE 3:** Differential Detection of CSF MiRNAs by TBI and Deployment Status.

	A. DepTBI vs. ComC		B. DepC vs. ComC		C. DepTBI vs. DepC	
miRNA	OR (CI)	*p*-value	OR (CI)	*p*-value	OR (CI)	*p*-value
127-3p	**2.35 (0.97 - 5.84)**	**0.0431**	3.41 (0.99 - 13.02)	0.0317	0.69 (0.18 - 2.43)	0.5790
362-3p	**8.90 (1.81 - 87.10)**	**0.0026**	3.06 (0.21 - 45.48)	0.2709	2.87 (0.54 - 29.42)	0.3143
152-3p	**5.68 (1.14 - 55.76)**	**0.0182**	Inf (0.96 - Inf)	0.0547	0.00 (0.00 - 13.46)	1.0000
502-3p	**3.62 (1.28 - 11.03)**	**0.0107**	1.10 (0.17 - 5.39)	1.0000	3.28 (0.76 - 20.17)	0.1375
548a-3p	**0.32 (0.12 - 0.80)**	**0.0107**	0.35 (0.10 - 1.22)	0.0781	0.91 (0.26 - 3.16)	1.0000
125a-5p	1.46 (0.59 - 3.67)	0.4068	**5.74 (1.16 - 56.57)**	**0.0207**	0.25 (0.03 - 1.32)	0.1155
362-5p	0.93 (0.30 - 2.80)	1.0000	**5.68 (1.59 - 22.00)**	**0.0029**	**0.16 (0.04 - 0.61)**	**0.0027**
548c-3p	1.21 (0.42 - 3.67)	0.8073	**0.18 (0.04 - 0.63)**	**0.0036**	**6.83 (1.79 - 29.36)**	**0.0024**

*MiRNAs with significant changes in detection in each group. (A). Bold font indicates miRNAs significantly differentially detected by TBI when comparing the DepTBI vs. ComC groups. (B). Bold font indicates miRNAs significantly differentially detected by deployment when comparing the DepC vs. ComC groups. (C). Bold font indicates miRNAs significantly differentially detected by TBI when comparing the DepTBI vs. DepC groups. Results include odds ratio (OR) with 95% confidence interval (CI), and p-value.*

### Detecting Multivariable Dependencies Among Biological Variables

We used an information theory-based method to systematically search through the study variables (such as miRNA expression levels, TBI status, BMI, etc.) and identify strong pairwise and 3-variable dependencies (Galas et al., 2014; Sakhanenko and Galas, 2015; Sakhanenko et al., 2017). Using our information measures, our data analysis pathway consisted of four main stages: preprocessing, information measure computation, output evaluation, and miRNA candidate selection (Figure 1).

#### Preprocessing: Binning and Sample Selection

We began by binning all continuous data such that it was represented by discrete and positive integers, a perquisite for calculating information measures. To preprocess miRNA expression levels we examined the distributions of values and discretized them into three bins using μ±*f*×*s* as thresholds, where μ is the mean, *s* is the standard deviation, and *f* is the factor, determined for each miRNA individually to ensure that the number of values in each bin are close to being equal. Each bin was encoded with an integer. The factor values for each miRNA are in Supplementary Table 4.

We distinguished between two types of phenotypes, primary and secondary, and converted them into binned variables as shown in Supplementary Table 8. For clarity, the binned variables (the integer-valued phenotypes constructed to carry out the information theory-based analysis) are in *lower case italics*, and we use *ptau* and *abeta* (without subscripts) to refer to the binned version of phenotypes pTau_181_ and Abeta_42_ (Supplementary Table 8). There are three primary binned variables representing the sample groupings based on deployment status and TBI history. The 2-value *deployment* variable considers ComC vs. combined DepC and DepTBI; the 2-value *tbi* variable considers combined ComC and DepC vs. DepTBI; and the 3-value *exp_grp* considers ComC, DepC, and DepTBI individually. The per-sample definitions of the three primary variables are shown in Supplementary Table 1.

All other phenotypes were considered secondary (Supplementary Table 8). Of the continuous phenotypes, Age and BMI were each assigned to three bins as for the miRNAs, resulting in binned variables *age* and *bmi*. The remaining continuous variables were discretized into two bins by splitting on the mean value to increase the number of samples per bin. Categorical phenotypes (*APOE* genotype, smoking status, race) were encoded using a 1-to-1 mapping into integers. In cases when the number of categories was more than 3, categories were merged to raise the number of samples in each bin. For example, the *APOE* genotypes (e2, e3, e4) in our data set were represented with a binary variable *apoe4* indicating whether or not the *APOE* genotype contains the e4 allele.

Not all phenotypes contained values in every sample (see ‘Number of Samples’ column in Supplementary Table 8). To avoid limiting the computation with missing data or dropped samples, we assembled data into individual input files for each type of dependency computation, therefore increasing the number of samples available for each computation.

#### Information Measurement: Calculating Mutual Information and Delta Scores

We next aimed to identify which of the 377 miRNAs on the array card were strongly dependent on the primary phenotypes (*deployment, tbi*, or *exp_group)* and secondary phenotypes (*age, bmi, mmse, abeta, ttau, ptau, abeta-ttau, abeta-ptau, ptau-ttau, apoe, apoe4, smoke*, and *race*) of study participants. We systematically searched for pairwise dependencies of the type ⟨*m*,*p*⟩ and for 3-variable dependencies of the type ⟨*m*,*p*,*s*⟩, where *m* represents an miRNA, *p* represents a primary phenotype, and *s* represents a secondary phenotype. For each given combination of *p* and *s* phenotypes, we calculated an information and delta score for each miRNA and selected top miRNAs based on their score, as described below.

First, in order to measure pairwise dependence between two variables, *M* and *P*, we used *mutual information I*(*M*,*P*), defined as


I(M,P)=H(M)+H(P)-H(M,P),

where *H*(*M*) and *H*(*P*) are single entropies of variables *M* and *P* and *H*(*M*, *P*) is their joint entropy.

In order to measure dependence between three variables, *M, P*, and *S*, we used *symmetric delta*, $\bar{\Delta }\left(M,P,S\right)$. To provide the definition of symmetric delta we introduce *interaction information*, which is a multivariable generalization of mutual information (McGill, 1954), defined for three variables as


I(M,P,S)=I(M,P)-I(M,P|S).

Given interaction information, we defined *differential interaction information* (Δ) as a difference between values of successive interaction information arising from adding a variable:


$$\begin{array}{c} \Delta_{M}=I\left(M,P,S\right)-I\left(P,S\right),\\ \Delta_{p}=I\left(M,P,S\right)-I\left(M,S\right),\\ \Delta_{S}=I\left(M,P,S\right)-I\left(M,P\right). \end{array}$$

Here Δ_*M*_ is called *asymmetric delta* for the target variable *M*. In order to detect a fully synergistic dependence among a set of variables, we want a single measure to be symmetric. Consequently, we defined a general measure $\bar{\Delta }$, called *symmetric delta* (or simply *delta*), by multiplying with all possible choices of the target variable:


$$\bar{\Delta }\left(M,P,S\right)=\Delta_{M}\cdot \Delta_{p}\cdot \Delta_{S}.$$

The critical property of the delta measure is that it is zero whenever any of the three variables is independent of the others. It is important to note that high absolute values of the delta measure or mutual information indicate that the corresponding variables are collectively interdependent. Conversely, small values of delta and mutual information indicate that variables are approximately independent. Note that both measures are symmetric under permutation of variables. Examples of two distributions, values of mutual information and delta measures, are shown in Supplementary Figure 1. By our definition, values of 3-variable delta are negative, while values of mutual information are always positive. Mutual information and delta can detect not only linear correlations, but any nonlinear relationships among variables.

#### Output Evaluation: Pairwise and 3-Variable Dependencies

We searched for dependencies between miRNA and participant phenotypes in two ways: firstly through pairwise dependencies ⟨*m*, *p*⟩ between a miRNA *m* and a primary phenotype *p*; and secondly through 3-way dependencies ⟨*m*, *p*, *s*⟩ of an miRNA *m*, a primary phenotype *p*, and a secondary phenotype *s*.

In the first search strategy, we used mutual information scores to identify miRNAs with the largest amount of information, which reduces uncertainty about the corresponding primary phenotype (e.g., when a miRNA and the phenotype are correlated). In the second search strategy, we used delta scores to identify miRNAs with the largest amount of 3-way information, which is shared with a primary and a secondary phenotype (knowing the secondary phenotype reduces uncertainty about the corresponding primary phenotype). In this strategy, the secondary phenotype can be viewed as a confounding factor affecting the dependency between an miRNA and a primary phenotype. Additionally, to capture the collective 3-way dependence between a miRNA, deployment, and tbi, we also searched for dependencies ⟨*miRNA*, *deployment*, *tbi*⟩, focusing only on the 3-way collective component of the relationship between these three variables.

During every search, we scored all 377 assayed miRNAs to provide a background signal for the analysis. We used an information measure (either mutual information or 3-variable delta) and selected up to 20 top-scoring miRNAs, whose scores were greater than one standard deviation away from the mean (see Supplementary Figure 1 for examples of score distributions). This resulted in 43 lists of pairwise and 3-way dependencies. 224 miRNAs (of the 377 assayed) appeared in at least one list of strong dependencies. Z-scores of information measures evaluated here for the 125 miRNAs that also passed the qPCR quality thresholds are shown in Supplementary Table 9.

#### Candidate Selection: Composite Scores

The scores between different searches cannot be directly compared, due to the difference between phenotype value distributions, a variable and small number of samples in each search, and the difference between pairwise and 3-variable measures. Consequently, we converted the raw information scores into *z*-scores, in order to compare them and to offer evidence of significance of the results. To narrow down the list of implicated miRNAs, we computed 3 composite scores corresponding to each of the three primary phenotypes (Supplementary Table 9). The composite score aggregates the evidence about a miRNA across all phenotypes of interest to measure the potential importance of that miRNA to that primary phenotypic grouping. For example, the *tbi* composite score is defined as:


$$\begin{array}{c} cs\left(tbi,m\right)=MI\left(tbi,m\right)+\frac{1}{2}(\max(\bar{\Delta }\left(tbi,ptau\text{-}ttau,m\right),\\ \bar{\Delta }\left(tbi,abeta\text{-}ttau,m\right),\bar{\Delta }\left(tbi,abeta\text{-}ptau,m\right))+\\ \Delta \left(tbi,apoe4,m\right))+\frac{1}{3}(\bar{\Delta }\left(tbi,age,m\right)+\bar{\Delta }\left(tbi,bmi,m\right)\\ +\bar{\Delta }\left(tbi,smoke,m\right)) \end{array}$$

Here, *m* is a miRNA, *MI* and $\bar{\Delta }$ are *z*-scores of the corresponding mutual information and delta values, and *tbi*, *ptau-ttau*, *abeta-ttau*, *abeta-ptau*, *apoe4*, *age*, *bmi*, *smoke* are variables corresponding to the binned phenotypes TBI, pTau_181_:tTau, Abeta_42_:tTau, Abeta_42_:pTau_181_ ratios, binary *APOE*, age, BMI, and current tobacco use (see Supplementary Table 8). Since phenotypes *ptau-ttau*, *abeta-ttau*, and *abeta-ptau* are correlated, only the maximal *z*-score was selected. Since the mutual information scores have less statistical fluctuation on a small sample set when compared to the delta scores, and since the delta scores tend to be more extremely distributed than the mutual information scores, we downgraded the delta scores by a factor of 2. This factor was further increased to 3 for the phenotypes *age*, *bmi*, and *smoke*, since the effects of these phenotypes on TBI is anticipated to be small.

### Target Prediction

To predict biological targets of differentially expressed miRNAs, we used our previously published bioinformatics pipeline (Sandau et al., 2020a) that utilizes the online prediction tools TargetScan v.7.2 and miRDB, followed by QIAGEN Ingenuity Pathway Analysis^2^. In order to specifically examine the effect of TBI on miRNA signaling, we analyzed the 10 miRNAs whose expression levels were significantly different in DepTBI vs. ComC (Table 2A). We also analyzed the 7 miRNAs whose expression levels were significantly different in DepC vs. ComC (Table 2B), and the 8 miRNAs whose expression levels were significantly different between DepTBI vs. DepC (Table 2C). We used TargetScan (Agarwal et al., 2015) and miRDB (Liu and Wang, 2019; Chen and Wang, 2020) to generate a list of mRNAs that are predicted targets of these miRNAs. These tools were chosen as they are both commonly used and frequently updated. In order to limit our list to targets that have been either experimentally validated or predicted with a high degree of confidence, targets were excluded if they had a Cumulative Weighted Context Score > −0.3 in TargetScan, or a Target Score < 80 in miRDB. The lists of mRNAs predicted by TargetScan (Supplementary Table 12) and miRDB (Supplementary Table 13) for all 3 datasets were then used in separate IPA analyses to identify putatively affected canonical pathways based on either TargetScan (Supplementary Table 14) or miRDB (Supplementary Table 15). Significant canonical pathways were based on adjusted p-values using a Benjamini-Hochberg false discovery rate threshold of 0.1 (Supplementary Table 16). In order to avoid the knowledge bias towards cancer in IPA, we excluded cancer-related tissues, cell lines, and diseases from this analysis.

## Results

### Participant Characteristics

Of the 115 participants in this study, 52 were in the ComC group, 18 were in the DepC group, and 45 were in the DepTBI group (Table 1 and Supplementary Table 1). All participants were male. The average age at CSF collection of the ComC group was 33.4 ± 9.3, of the DepC group was 31.8 ± 7.1, and the DepTBI group was 34.0 ± 10.0 (ANOVA *p* = 0.71). BMI of the ComC group was 25.1 ± 3.4, of the DepC group was 26.9 ± 4.3, and the DepTBI group was 28.5 ± 4.4 (ANOVA *p* < 0.001); post hoc testing showed that the DepTBI group had significantly higher BMI than ComC (*p* < 0.001). MMSE of the ComC group was 29.5 ± 0.6, of the DepC group was 29.1 ± 1.1, and the DepTBI group was 28.5 ± 1.6 (ANOVA *p* = 0.0003); post hoc testing showed that the DepTBI group had significantly lower MMSE than ComC (*p* < 0.001). The number of reported mTBIs in the DepTBI group was 20.5 ± 28.9 [1 – 102]. The average time since the last blast mTBI experienced was 4.7 ± 2.2 years [1.5 – 11.5]. The CSF Abeta_42_:tTau measures in the ComC group were 8.2 ± 2.1, of the DepC group was 9.4 ± 1.9, and the DepTBI group was 9.1 ± 2.1 (ANOVA *p* = 0.067). The CSF pTau_181_:tTau measures in the ComC group were 0.68 ± 0.15, of the DepC group were 0.84 ± 0.10, and in the DepTBI group were 0.80 ± 0.14 (ANOVA *p* < 0.001); post hoc testing showed that the DepC and the DepTBI groups each had significantly higher pTau_181_:tTau ratios than ComC (DepC *p* < 0.01, DepTBI *p* < 0.01).

### Differential Expression Levels of MiRNAs in CSF by TBI and Deployment Status

**FIGURE 2:**
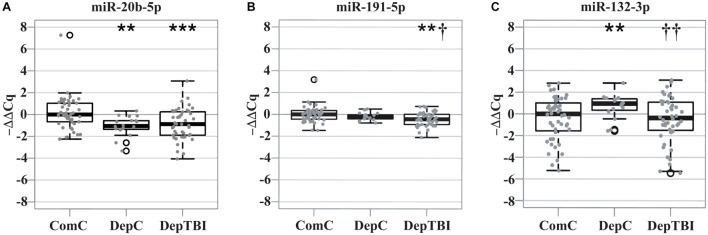
Distribution of –ΔΔCq Values for Representative MiRNAs in Each Group. Box plots show the distribution of the –ΔΔCq values for representative miRNAs differentially expressed with respect to ComC in Table 2. The plots show that **(A)** miR-20b-5p is significantly altered in the DepC and DepTBI groups **(B)** miR-191-5p is significantly altered in the DepTBI group, and **(C)** miR-132-3p is significantly altered in the DepC group. Significance: **p < 0.01, ***p < 0.001 with respect to ComC, ^†^ *p* < 0.025, ^††^ *p* < 0.01 with respect to DepC.

We identified 134 miRNAs in CSF that passed our quality control criteria (see section “MATERIALS AND METHODS”). Of these we compared the levels between groups to identify miRNAs that are differentially expressed in DepTBI vs. ComC, DepC vs. ComC, and DepTBI vs. DepC. All results for the differential levels of miRNA by experimental group comparisons are listed in Supplementary Table 5. These analyses revealed that there was an effect of both TBI and deployment status on CSF miRNA levels. Fold changes were considered significant if they had a *p*-value < 0.025. We identified a total of 18 differential miRNAs. Ten were significantly different between DepTBI and ComC (Table 2A); 7 between DepC and ComC (Table 2B); and 8 between DepTBI and DepC (Table 2C). Table 2 shows that 2 miRNAs were differentially expressed in both DepTBI and DepC vs. ComC (miR-548-3p, miR20b-5p); 1 miRNA was differentially expressed in DepTBI vs. ComC and DepTBI vs. DepC (miR-191-5p); and 4 miRNAs were differentially expressed in both DepC vs. ComC and DepTBI vs. DepC (miR-152-3p, miR-132-3p, miR-362-5p, miR-548c-3p). Figure 2A shows that miR-20b-5p has significantly lower expression in DepC (*p* < 0.01), and in DepTBI (*p* < 0.001) vs. ComC. Figure 2B shows that miR-191-5p is significantly lower in DepTBI (*p* < 0.01) vs. ComC, and in DepTBI vs. DepC, and Figure 2C shows that miR-132-3p is significantly increased in DepC (*p* < 0.01) vs. ComC.

### Differential Detection of CSF MiRNAs by TBI and Deployment Status

**FIGURE 3:**
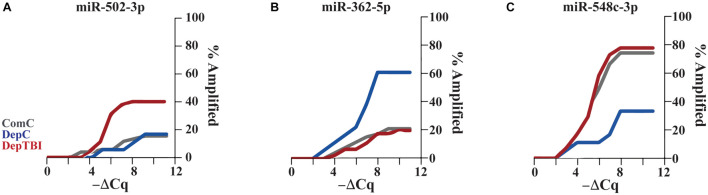
Time to Amplification Plots for CSF MiRNAs Differentially Detected in Each Group. The figures depict representative time to amplification plots for miRNAs with statistically significant changes in detection, *p* < 0.025. Representative plots show differential detection rates for **(A)** miR-502-3p that increased in the DepTBI vs. ComC and DepC group, **(B)** miR-362-5p that increased in the DepC group relative to both ComC and DepTBI, and **(C)** miR-548c-3p was decreased in DepC relative to both ComC and DepTBI.

Since several miRNAs were detected at different rates in a subset of samples, we utilized Fisher’s exact test to determine whether the detection rates might be useful to classify TBI status. All results for the differential detection rates of the 134 miRNAs found in CSF by experimental group comparisons are listed in Supplementary Table 6. For each miRNA differentially detected between groups, we calculated odds ratios and *p*-values; significance was set as *p* < 0.025 (Table 3). This analysis revealed that both TBI and deployment status are associated with the expression of miRNAs not commonly measured in the ComC group. Table 3 lists the 8 miRNAs with significant differentially detection in the group comparisons: 5 miRNAs were differentially detected in DepTBI vs. ComC (3A) (miR-127-3p, miR-362-3p, miR-152-3p, miR-502-3p, miR-548a-3p); 3 miRNAs were differentially detected in DepC vs. ComC (3B) (miR-125a-5p, miR-362-5p, miR-548c-3p); and 2 miRNAs were differentially detected in DepTBI vs. DepC (3C) (miR-362-5p, miR-548c-3p). Figure 3 shows representative time to amplification plots showing differential detection rates for (A) miR-502-3p that increased in DepTBI vs. ComC, (B) miR-362-5p that increased in DepC vs. ComC and DepTBI, and (C) miR-548c-3p that was decreased in DepC with respect to ComC and DepTBI.

### Effects of Participant Characteristics on Differential Expressed or Detected MiRNAs

For the miRNAs differentially expressed and/or detected, we examined correlations with age at CSF collection, MMSE scores, BMI, current tobacco use, number of TBIs, years since most recent TBI, Abeta_42_:tTau ratio, and pTau_181_:tTau ratio, Abeta_42_, tTau, and pTau_181_ levels to miRNA expression level for each participant (Supplementary Table 7). Using uncorrected *p*-values, we found correlations in each of the groups. For the DepTBI samples, we found a correlation with MMSE for miR-132-3p (*p* = 0.016), with current tobacco use and miR-127-3p (*p* = 0.006), and with Abeta_42_:tTau for miR-140-5p (*p* = 0.011). In the DepC samples, we found a correlation with age at sampling and miR-140-5p (*p* = 0.010). In the ComC samples, we found a correlation with age at sampling and miR-191-5p (*p* = 0.023) and pTau levels and miR-30b-5p (*p* = 0.011).

### MiRNA Verification Study

The initial “discovery” studies were performed on a commercially available card with *n* = 1 technical replicate probe per miRNA. For the verification studies we generated custom miRNA cards, with *n* = 3 technical replicate probes per miRNA, to examine miRNA expression in 30 CSF samples used in the initial studies (*n* = 10 / experimental group). We first assessed whether the measurements for each individual were well correlated across the discovery and verification assays. We found strong within participant correlation (relative expression of each miRNA in each individual) between miRNAs expressed in both the initial and verification study, i.e., median correlation 0.789; range 0.574 to 0.887 (Supplementary Table 10). We then assessed the ΔCq correlation for each miRNA between the discovery and verification assays (Supplementary Table 11). Here we focused on the miRNAs that were considered in the differential expression and detection analyses, to ensure they were well represented in each participant group. Two miRNAs (miR-518-3p and miR-92a) did not perform optimally in the verification study. Firstly, miR-518-3p, a miRNA differentially expressed in DepC vs. ComC, failed to amplify in the verification. Secondly, miR-92a, a positive control expressed in CSF that was not differently expressed between the groups, had a low correlation of 0.142 between the discovery and verification. The failed amplification for miR-518-3p and low correlation for miR-92a may reflect differences in probe performance between array lots, as we have observed in previous studies. Of the remaining miRNAs, 7 had correlation estimates ranging from 0.506 to 0.811 between the discovery and verification studies. MiR-152-3p showed a significant change in expression in the discovery study, and a correlation of 0.488 (*p* = 0.006) in the verification study. Furthermore, the negative control miR-409-5p (not expressed in CSF) was not detected in any of the 30 samples, while the positive control miR-204 had a correlation of 0.677. Together these results indicate that overall, data generated in the discovery study were reproducible.

### Multi Variable Dependence

For the information theory-based analysis we considered three primary phenotypes: (i) *deployment*, which considers ComC vs. combined DepC and DepTBI; (ii) *tbi*, which considers combined ComC and DepC vs. DepTBI; and (iii) *exp_grp* which considers ComC, DepC, and DepTBI individually. We further considered 13 secondary phenotypes: *age, bmi, mmse, abeta, ttau, ptau, abeta-ttau, abeta-ptau, ptau-ttau, apoe, apoe4, smoke, race*. Using information theory-based dependency analysis (Supplementary Figure 1) we searched for dependencies between 377 miRNAs and the phenotypes. The analysis resulted in 125 miRNAs that are involved in strong dependencies and passed the qPCR quality thresholds. The list of these miRNAs with their corresponding dependency scores, represented as *z*-scores, are in Supplementary Table 9. It is critical to recall that low *z*-scores of mutual information and delta measures indicate that variables are approximately independent, whereas high *z*-scores indicate that the corresponding variables are **collectively** interdependent. Furthermore, mutual information and delta can detect not only linear correlations, but any nonlinear relationships among variables.

**FIGURE 4:**
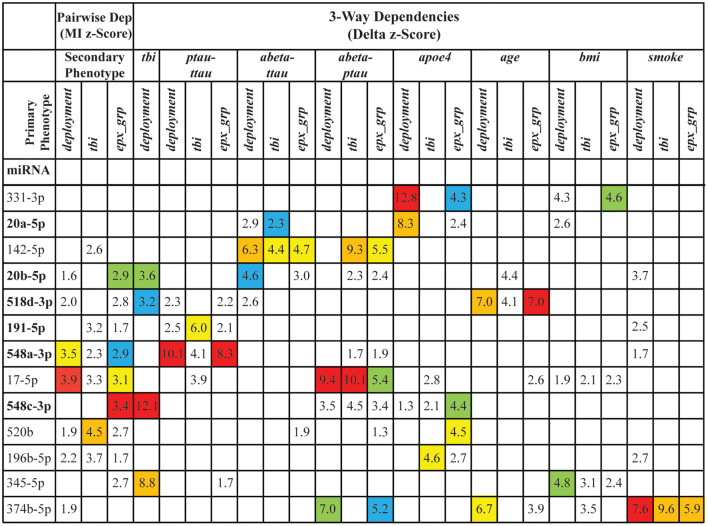
The stringent filtering of dependencies. The figure shows 13 miRNAs that passed the stringent filtering based on their composite scores. The 6 miRNAs in bold were also identified using *differential levels* and/or *differential detection*. The values in the cells are the *z*-scores of the corresponding pairwise dependencies (Dep) [based on mutual information (MI)] or 3-way (based on delta measures) dependencies. Empty cells indicate that the corresponding dependency was not a top dependency. Colors highlight the ranking of the dependency in each of the original lists of strong dependencies: red indicates the top dependency in the list, orange – the dependency ranked 2, yellow – 3, green – 4, and blue – 5. Note that the columns correspond to individual distributions of information scores, which are based on different phenotypes, and therefore the z-scores of the dependencies with the same rank across the columns are not similar.

To assess how informative a miRNA is about the primary phenotype, we derived a composite score from the mutual information and delta *z*-scores for each primary phenotype-miRNA pair. We then selected the secondary phenotypes that play important role in the development of AD, namely *apoe4*, *age*, *bmi*, *smoke*, *ptau-ttau*, *abeta-ttau*, *abeta-ptau*, and for each miRNA calculated three composite scores, one for each primary phenotype. Based on the distribution of all composite scores, we derived stringent filtering (Supplementary Table 9) focusing our attention only on miRNAs with the strongest dependencies (Figure 4).

To better understand the composite information scores (Figure 4), we need a closer look at the primary phenotypes. If we look at the primary phenotypes as vectors of values, assuming a certain order of samples, then *deployment*, *tbi*, and *exp_grp* are identical for all the samples from ComC and all the samples from DepTBI (see Supplementary Table 1). The difference between these three phenotypes comes from the way the samples from DepC were handled: in *deployment* samples were merged with the samples from DepTBI, in *tbi* samples were merged with the samples from ComC, and in *exp_grp* samples were left as a separate group resulting in 3-valued phenotype. Clearly these three phenotypes are correlated, but only partially, and the differences between these phenotypes could explain why there are miRNAs whose dependencies are stronger with one phenotype, but not with the other. For example, miR-17-5p is the strongest miRNA based on the strength of pairwise dependence with the primary phenotype *deployment* (rank = 1). Although still relatively strong, the miR-17-5p dependence with *tbi* is weaker (rank = 7). Additionally, the dependence of miR-17-5p with *exp_grp* (rank = 3) is between *tbi* and *deployment* rank wise. This suggests that miR-17-5p behavior in civilians is very different from that in veterans. Furthermore, it is the deployment and not the status of TBI that is the “driver” of this dependence.

A similar example is miR-520b, but this time it is strongly connected with *tbi* (the highest rank among all considered miRNAs) and weakly connected with *deployment* and *exp_grp* (ranked 15 and 8 correspondingly). This suggests that miR-520b behavior changes considerably when we go from the DepTBI samples to the remaining samples.

Although miR-548c-3p is not ranked in either *deployment* or *tbi*, it is ranked the highest in *exp_grp*. This suggests a collective dependence among these 3 variables, which is further validated from the 3-way analysis of miRNAs together with *deployment* and *tbi*, where miR-548c-3p ranked the highest. This means that knowing the level of miR-548c-3p alone is not enough to significantly reduce our uncertainty about *tbi*, but knowing both the miRNA level and *deployment* together reduces the largest amount of uncertainty about *tbi*. MiR-345-5p, miR-20b-5p, and miR-518d-3p have a similar collective 3-way dependence with *deployment* and *tbi*. There are also strong collective, 3-way dependencies among miRNAs, the primary phenotypes, and the secondary phenotypes, involving both miRNAs that have strong pairwise dependence on the primary phenotype (such as miR-196b-5p and miR-548a-3p). Neither miR-331-3p nor miR-20a-5p depend on any primary phenotypes, but both demonstrate strong dependence on *apoe4.*

### MiRNA Target Prediction

**FIGURE 5:**
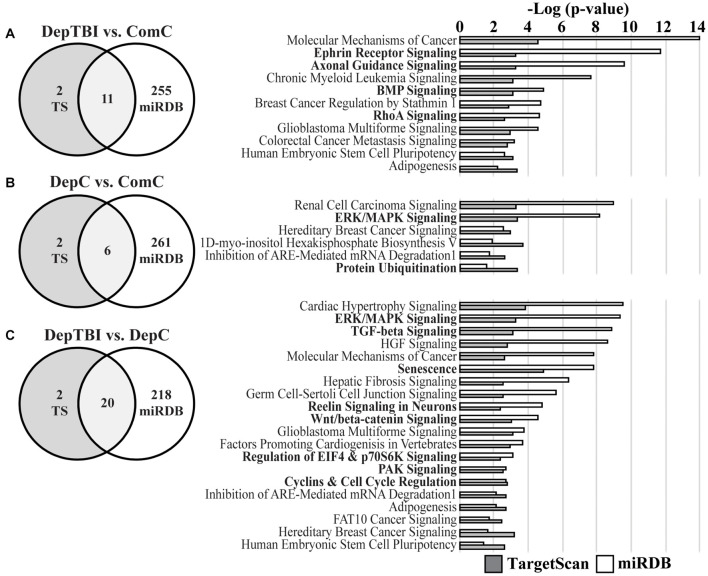
Top Canonical Pathways for Predicted mRNA Targets of MiRNAs with Differential Levels. Venn diagrams report the number of significant canonical pathways identified by IPA for mRNA targets predicted by TargetScan v.7.2 (TS) and miRDB (left) and the significant canonical pathways identified by both the TS (gray bars) and miRDB (white bars) mRNAs (right). **(A)** Target prediction and pathways analysis for miRNAs differentially expressed in DepTBI vs. ComC. Four of the significant pathways implicated in TBI are indicated by bold font. **(B)** Target prediction and pathways analysis for miRNAs differentially expressed in DepC vs. ComC. Two of the significant pathways implicated with the neurological effects of chronic stress are indicated by bold font. **(C)** Target prediction and pathways analysis for miRNAs differentially expressed in DepTBI vs. DepC. Eight of the significant pathways implicated in TBI and/or chronic stress are indicated by bold font. Significant canonical pathways identified by IPA were based on adjusted *p*-values using a Benjamini-Hochberg false discovery rate threshold of 0.1.

We specifically investigated the effect of TBI alone on miRNA signaling by focusing target prediction analysis on 10 miRNAs that showed significantly different expression levels between the DepTBI and ComC groups (Table 2A): miR-20b-5p, miR-548a-3p, miR-197-3p, miR-140-5p, miR-20a-5p, miR-191-5p, miR-362-3p, miR-502-3p, miR-30b-5p, miR-30c-5p. We also analyzed the 7 miRNAs whose expression levels were significantly different between DepC and ComC (Table 2B): miR-548a-3p, miR-20b-5p, miR-152-3p, miR-132-3p, miR-362-5p, miR-518d-3p, miR-548c-3p, and the 8 miRNAs whose expression levels were significantly different between DepTBI and DepC (Table 2C): miR-191-5p, miR-152-3p, miR-132-3p, miR-362-5p, miR-548c-3p, miR-125a-5p, miR-130a-3p, miR-411-5p. For the miRNAs in DepTBI vs. ComC (Table 2A), TargetScan predicted 684 mRNA targets, while miRDB predicted 2035 mRNAs (Supplementary Tables 12, 13). We then used the two lists of predicted mRNAs in separate IPA analyses to identify canonical pathways. For the miRNAs identified in DepTBI vs. ComC, the predicted mRNA targets were implicated in 13 and 266 canonical pathways, in TargetScan and miRDB, respectively (Supplementary Tables 14, 15), with an overlap of 11 canonical pathways (Figure 5A; Supplementary Table 16). Four of these pathways have been implicated in TBI: ephrin receptor signaling, axonal guidance, BMP (bone morphogenetic protein), and RhoA signaling. For the miRNAs in DepC vs. ComC (Table 2B), TargetScan predicted 196 mRNA targets, while miRDB predicted 1550 mRNAs (Supplementary Tables 12, 13). We again used the two lists of predicted mRNAs in separate IPA analyses to identify canonical pathways. The predicted mRNA targets were implicated in 8 and 267 canonical pathways, in TargetScan and miRDB, respectively (Supplementary Tables 14, 15) with an overlap of 6 canonical pathways (Figure 5B; Supplementary Table 16). Two of these pathways have been implicated in chronic stress: ERK/MAPK signaling and protein ubiquitination. For the miRNAs in DepTBI vs. DepC (Table 2C), TargetScan predicted 481 mRNA targets, while miRDB predicted 1279 mRNAs (Supplementary Tables 12, 13). We then used the two lists of predicted mRNAs in separate IPA analyses to identify canonical pathways. The predicted mRNA targets were implicated in 22 and 238 canonical pathways, in TargetScan and miRDB, respectively (Supplementary Tables 14, 15) with an overlap of 20 pathways (Figure 5C; Supplementary Table 16). Eight of these pathways have been implicated in TBI: ERK/MAPK, TGF-β, senescence, reelin signaling in neurons, Wnt/β-catenin, eIF4 and p70S6K, PAK, and cyclins and cell cycle regulation.

## Discussion

The overall goal of this study was to identify whether CSF miRNA levels might reflect a history of mTBI in otherwise healthy individuals. We hypothesized that these altered miRNAs may reflect biological changes in the brain that lead to increased risk for developing AD later in life. We assayed miRNA levels in three groups: community controls (ComC), deployed controls (DepC), and deployed TBI (DepTBI). Individuals in the control groups had no lifetime history of TBI. We used three approaches to assess changes in miRNA levels in this study: one based on statistics, one based on information theory, and another based on miRNA target prediction. Using the statistics approach we identified miRNAs in CSF that exhibited either differential expression levels (Table 2) or were differentially detected (Table 3) across the three groups. Most of the miRNAs showing differential levels were decreased in the deployed groups; 7 decreased in DepTBI, 2 decreased in both DepTBI and DepC, and 2 decreased in only DepC. However, there was 1 miRNA increased in DepTBI, and 3 miRNAs increased in DepC. Since a large fraction of miRNAs were not detected in all of the samples, we performed a differential detection analysis that identified 4 miRNAs preferentially expressed in DepTBI vs. ComC, and 3 miRNAs between DepC and ComC groups. Using information theory, we identified 13 miRNAs that vary with deployment and/or TBI history alone, or in combination with serum proteins, *APOE* genotype, age, BMI, or smoking. These additional phenotypes are potential confounding factors that may influence miRNA measurements in this study. Six miRNAs were identified in both the statistics and information theory analyses. We performed target prediction analysis on the miRNAs that showed significantly different levels between the three groups (Table 2) using TargetScan and miRDB, followed by IPA analyses of each target prediction. The motivation for this study was the hypothesis that miRNAs in the TBI group would be most distinct from the other two groups. Consistent with this, in the statistical analysis we found miRNAs that were significantly increased in expression in the DepTBI vs. ComC and DepTBI vs. DepC groups, but not in DepC vs. ComC, that reflect changes specific to TBI (e.g., miR-191-5p). However, we also found miRNAs that were changed in both the DepTBI and DepC groups that likely represent deployment-sensitive miRNAs (e.g., miR-548a-3p, miR-20b-5p). The effect of deployment upon miRNA expression was not unexpected, and may reflect environmental stressors and exposures specific to deployment. For example, both the DepC and DepTBI groups were exposed to environmental toxins from burn pits, and commonly used insecticides in the war zone (Dalgard et al., 2016; Woeller et al., 2016). Of the 6 overlapping canonical pathways identified by IPA in DepC vs. ComC, 2 of these pathways have been associated with the neurological effects of chronic stress: ERK/MAPK signaling and protein ubiquitination (Figure 5B; Ferland et al., 2014; Yuen et al., 2017).

It is important to note that previous studies of CSF miRNAs in TBI and in AD have largely examined participants at acute times after TBI (<4 weeks), or participants with AD, whereas our study captures a chronic post-TBI time in participants who were cognitively normal at the time. Thus, many of the previously reported miRNAs for TBI or AD would not be expected to be altered in the healthy participants studied here. For example, acute changes in blood miR-146a levels are detected in patients with severe TBI after injury at high altitude, and downstream gene targets including regulators of inflammatory responses, apoptosis, and DNA damage/repair (Ma et al., 2019). In our previous studies we identified miR-146a, a marker of neuroinflammation (Gaudet et al., 2018; Slota and Booth, 2019), as a potential biomarker for AD (Lusardi et al., 2017; Wiedrick et al., 2019). However, in the current study miR-146a showed no sensitivity for deployment or TBI, which may be due to the TBI severity for individuals in this study, or may reflect the transient nature of a miR-146a response to TBI.

Heterogeneity of the participants in this study may have affected the outcome of the differential levels and detection analyses. Although the study population was carefully defined as neurologically normal without significant cognitive impairment at the time of CSF collection, with strict exclusion criteria (history of moderate or severe TBI, substance abuse, or medications that may alter cognitive performance), we relied on self-reported history of TBI using careful clinical semi-structured interview performed by two expert TBI clinicians simultaneously. As this interview covered both the deployment and pre-deployment time frames, the data may suffer from a recall bias, which is unavoidable for the era prior to widespread use of electronic medical records. While the TBI-related inclusion characteristics were stringently applied, there was significant heterogeneity in associated participant characteristics. For example, in the TBI group, age at CSF collection varied across a 20 year span, the number of reported injuries varied from 1 to 102, and the time between most recent TBI and CSF collection varied from 1.5 to 11.5 years. In addition, approximately half of the DepC group served in combat military occupational specialties, while the rest were administrative or support personnel. Thus, significant differences in the miRNAs of the DepC group may reflect the small DepC sample size, or other factors not captured in this study.

Beyond simply measuring miRNA levels, biomedical data is filled with various dependencies since it is obtained from complex systems with many interactions. Hence, we used more sophisticated methods to detect multivariable dependencies of diverse kinds in order to effectively analyze the biological data. We recently introduced an information theory-based set of dependency measures and an approach to discover multivariable dependencies in a large set of variables capitalizing on a distinct advantage of separating the detection of the dependence from defining the nature of the dependence (Galas et al., 2014; Sakhanenko and Galas, 2015; Sakhanenko et al., 2017). In general, information theory measures have several advantages: they are inherently model-free and non-parametric in nature, and they exhibit only modest sensitivity to undersampling (McGill, 1954). Therefore, this method is a good fit for the present study, where the data set is relatively modest, and is quite variable in the phenotypes that might be expected to influence CSF miRNA levels such as age, BMI, or smoking.

While our methods can analyze any number of interacting variables, we are limited by sample numbers and, thus, considered only pairwise and three-way dependencies. We used the three-way dependence method to discover miRNAs that were simultaneously interdependent with two phenotype variables, one each from the primary and secondary phenotype sets. The use of our three-variable dependency measure has been shown to yield a number of interesting results that could not be detected using only two-way methods (Sakhanenko and Galas, 2015), which has significant implications for the way in which human biomedical data are analyzed. As a result, some miRNAs are implicated in three-way dependencies only and are not discovered by any pairwise dependence.

Although information measures do not give us insight into cause or effect of relative abundance changes, they are useful for identification of confounding or contributing factors to an outcome. Using information measures, we identified phenotypes that might be either positively or negatively reflected in the miRNA levels. We show that several miRNAs have high information scores with known AD-relevant CSF protein biomarkers Abeta_42_, tTau, and pTau_181_ (miR-191-5p, miR-20a-5p, miR-140-5p, miR-30c-5p, and miR-362-5p), though this analysis cannot demonstrate how or whether these miRNAs are functionally related to these protein biomarkers. In addition, age has high information scores with miR-30c-5p, BMI with miR-20a-5p, and smoking with miR-161-5p, suggesting that these are all relevant phenotypic information that should be collected and evaluated in follow up studies. Notably, none of these relationships were identified in the isolated correlation analyses (e.g., miRNA correlation to an individual phenotype in Supplementary Table 7).

The current study was done to examine whether there were changes in miRNAs in the chronic post-TBI state that might underlie progression to ADRD. Several published studies have reported a link between a history of brain injury and ADRD risk in civilian settings such as in professional football players (Guskiewicz et al., 2005), which led the National Football League to warn players of possible long-term health effects of concussions (DeKosky et al., 2010). Relevant to the current study, retrospective cohort studies have demonstrated increased risk of dementia in veterans, including two which represented large cohorts of veterans. One study that included 188,764 United States veterans aged 55 years or older, who had at least one inpatient or outpatient visit during both the baseline (2000-2003) and follow-up (2003-2012) periods and no dementia diagnosis at baseline, revealed that TBI in older veterans was associated with a 60% increase in the risk of developing dementia over the 9 year study period, after accounting for competing risks and potential confounders (Barnes et al., 2014). Another study on >350,000 United States Iraq/Afghanistan Veterans revealed that even mTBI without loss of consciousness was associated with >2 fold increase in risk for dementia diagnosis (Barnes et al., 2018). A smaller study evaluated the association between early adult head injury (documented by military records) and dementia in late life in World War II United States Navy or Marine male veterans who served during 1944 to 1945. Of 548 veterans with head injury and 1228 without head injury, the authors found that both moderate and severe head injury were associated with increased risk of AD (Plassman et al., 2000).

The first population-based study that investigated the association between TBI and the risk of young onset dementia (dementia before 65 years of age) focused on a cohort comprised 811,622 Swedish men (mean age 5-18 years) conscripted for military service between 1969 and 1986 (Nordstrom et al., 2014). The study revealed strong associations between young onset dementia of non-AD forms and TBIs of different severity. However, these associations were markedly attenuated after multivariate adjustment for low socioeconomic status, alcohol intoxication, physical fitness, blood pressure, and low premorbid cognitive function (Nordstrom et al., 2014). A follow-up study on all inhabitants living in Sweden over 50 years of age examined three cohorts for dementia: (1) 164,334 individuals with TBI matched with controls; (2) 136,233 individuals with dementia matched with controls; and (3) 46,970 full sibling pairs discordant for TBI. The risk of dementia was increased by four to six times the first year after TBI, but thereafter the risk decreased rapidly and was still significant more than 30 years after the TBI. Furthermore, the risk of dementia was higher for those with a severe TBI or multiple TBIs, compared to those with one mTBI (Nordstrom and Nordstrom, 2018). While the Nordstrom studies above focused on men (2014) as well as men and women (2018), one study reported that women with military-related risk factors had an ∼50% to 80% increase in developing dementia, and that female veterans with multiple risk factors had a >2-fold risk of developing dementia (Yaffe et al., 2019).

It is important to note that not all studies have demonstrated a strong link between mTBI and risk for dementia. In 2013, members of the Departments of Defense and Veterans Affairs joined a meeting hosted by the Alzheimer’s Association in order to strategize about research partnerships to move the field forward and address these discrepancies (Weiner et al., 2013). Participants at the “Military Risk Factors for AD” meeting had not traditionally collaborated or considered related mechanisms and markers of disease, but they did agree that coordinated efforts were required as a first step toward fully understanding the relationship of TBI (and post-traumatic stress disorder) to AD. A subsequent report stressed that epidemiologic studies of the relationship between TBI and AD suffered from two major limitations: (1) most used self-report information to determine a history of TBI exposure; and (2) most relied on billing codes for clinical diagnosis of AD dementia rather than using an array of recently developed biomarkers or neuropathologic examination (Weiner et al., 2017a). The report, titled “Traumatic brain injury may not increase the risk of Alzheimer disease” cites three large, well-powered, and carefully conducted studies that cast substantial doubt on the association between TBI exposure and AD outcomes, both overall and among men and carriers of *APOE4* alleles (Gardner et al., 2015; Crane et al., 2016; Weiner et al., 2017b). Thus, these studies highlight the complexity of the issue in question: Does head injury (TBI) initiate events that lead to increased risk of dementia (ADRD), which may now be resolved by studies that include newer and more sophisticated clinical measures (imaging, biomarkers) of TBI and ADRD?

To put the present findings from this study into context, we compared the miRNAs identified in this study to primary studies in patients that had a recent TBI (Kulbe and Geddes, 2016; Pan et al., 2017; Di Pietro et al., 2018; Atif and Hicks, 2019), or to confirmed human AD cases (Dorval et al., 2013; Zafari et al., 2015; Kumar and Reddy, 2016; Putteeraj et al., 2017; Herrera-Espejo et al., 2019). Of the miRNAs identified in this study that show differential levels (Table 2) or differential detection (Table 3) at a post-acute time following TBI, most have been linked to acute TBI and/or AD in previously published studies. Table 4 includes the primary references for each of these studies, to maintain ease of reading here. In short, we found that: (1) only miR-502-3p had been previously implicated in acute TBI, but not AD; (2) miR-132-3p, miR-152-3p, miR-191-5p, miR-20a-5p, miR-30b-5p, miR-362-3p, and miR-345-5p had previously been implicated in both acute TBI and AD, and (3) miR-125a-5p, miR-140-5p, miR-197-3p, miR-20b-5p, miR-30c-5p, miR-362-5p, miR-331-3p, miR-142-5p, miR-17-5p, and miR-374b-5p had previously been implicated in AD only. Our findings also overlap with a published meta-analysis, which integrated data from 107 publications on miRNA expression in AD brain, serum, saliva using various methods (TLDA, RNASeq, NanoString, etc.) which found 57 miRNAs with study-wide significance (Takousis et al., 2019). Of these 57, miR-191-5p (Table 2), miR-127-3p (Table 3), and miR-152-3p (Table 3) are in common with miRNAs identified in previously published AD studies (Takousis et al., 2019). In addition, a recent study measured miRNAs in plasma from a subset of participants from the CSF cohort herein, both studies drew CSF samples from an ongoing longitudinal multimodal assessment study of veterans with and without repetitive mTBI (Petrie et al., 2014). The plasma study identified 32 miRNAs that were significantly increased in mTBI vs. community controls and deployed controls (Ghai et al., 2020). Of the 32 plasma miRNAs increased in TBI, only miR-20a-5p and miR-20b-5p (Table 2) were altered in CSF, but were significantly decreased in TBI. These differences in study outcomes likely represent the differences in plasma vs. CSF miRNAs, or in the number of overlapping CSF vs. plasma participants in each study: ComC 52 vs. 25, DepC 18 vs. 11; DepTBI 45 vs. 24.

**TABLE 4:** Evidence Linking MiRNAs Altered by Post-Acute TBI to Acute TBI and/or AD.

miRNA	Acute TBI	Post-Acute TBI	AD
502-3p	Down Serum (Di Pietro et al., 2017)	Up vs. ComC	
132-3p	Up Serum (Di Pietro et al., 2017)	Down vs. DepC	Up serum (Denk and Jahn, 2018) Down HP, MFG (Cogswell et al., 2008) Down HP, PFC (Lau et al., 2013) Up CSF (Burgos et al., 2014)
152-3p	Up Serum (Di Pietro et al., 2017)	Up vs. ComC; Down vs. DepC	Down Plasma Exosomes (Lugli et al., 2015)
191-5p	Up Serum (Yang et al., 2016)	Down TBI vs. ComC, DepC; *tbi × ptau-ttau*	Up, CSF (Cogswell et al., 2008) Up Plasma (Kumar et al., 2013) Down Serum (Tan et al., 2014)
20a-5p	Up Serum (Bhomia et al., 2016); Up Saliva, (LaRocca et al., 2019)	Down TBI vs. ComC; *tbi × abeta-ttau; dep × apoe4*	Down, Serum (Denk and Jahn, 2018) Up Serum Exosomes (Cheng et al., 2015)
30b-5p	Up CSF (You et al., 2016); Up Saliva and Serum (LaRocca et al., 2019)	Down TBI vs. ComC	Up Serum (Denk and Jahn, 2018) Increase CSF (Cogswell et al., 2008) Correlations: CSF and Tangles, Serum and Plaques (Burgos et al., 2014)
362-3p	Up Serum (Bhomia et al., 2016)	Mixed vs. ComC	Down HP (Lau et al., 2013)
125a-5p		Down vs. DepC	Up CSF (Denk and Jahn, 2018)
140-5p		Down vs. ComC	Down CSF (Lusardi et al., 2017; Wiedrick et al., 2019; Sandau et al., 2020b)
197-3p		Down vs. ComC	Up Serum (Denk and Jahn, 2018) Up CSF (Cogswell et al., 2008)
20b-5p		Down vs. ComC; *exp_grp; dep × tbi*	Up HP APPswe/PSΔE9 Mouse (Tian et al., 2021)
30c-5p		Down vs. ComC	Up CSF (Cogswell et al., 2008)
362-5p		Down vs. DepC	Up CSF (Cogswell et al., 2008)
127-3p		Up vs. ComC	Down CSF (Cogswell et al., 2008) Down PFC (Lau et al., 2013) Up CSF, Down Serum (Burgos et al., 2014)
345-5p	Up Serum (Di Pietro et al., 2017)	*dep × tbi; dep × bmi*	Up CSF (Cogswell et al., 2008)
331-3p		*dep × apoe4; exp_grp × apoe4; exp_grp × bmi*	2021 Down Serum (Liu and Lei, 2021)
142-5p		*dep × abeta-ttau; tbi × abeta-ttau; exp_grp × abeta-ttau; tbi × abeta-ptau; exp_grp × abeta-ptau*	Down CSF (Cogswell et al., 2008) Up PFC (Lau et al., 2013)
17-5p		*dep; exp_grp; dep × abeta-ptau; tbi × abeta-ptau; exp_grp × abeta-ptau*	Down PFC (Lau et al., 2013)
374b-5p		*dep × abeta-ptau; exp_grp × abeta- ptau; dep × age; dep × smoke; tbi × smoke; exp_grp × smoke*	Up PFC (Lau et al., 2013) Up Serum (Burgos et al., 2014)

*CSF miRNAs identified in post-acute TBI with significant differential levels or differential detection with respect to ComC or DepC, or in the top 5 ranked Information Theory dependencies that have been reported in primary publications on human miRNA expression in acute TBI (<4 weeks) and/or in AD. miR-362-3p is designated “mixed” as it is detected in more DepTBI samples than in ComC, but at lower levels. HP, hippocampus; MFG, Medial Frontal Gyrus; PFC, Prefrontal Cortex.*

We also examined the potential mechanistic implications of the miRNAs in Table 4 by reference to previously published studies. A recent comprehensive review by Wang et al., reported the mechanistic effects of miRNAs on proteins involved in different pathological processes of AD progression, including Abeta, beta-secretase (BACE), amyloid precursor protein (APP), and tau (Wang et al., 2019). Studies show that miR-132/212 (miR-132-3p; Table 2) indirectly regulate Abeta_40_ and Abeta_42_ (Hernandez-Rapp et al., 2016) and tau (Smith et al., 2015; Wang et al., 2017; El Fatimy et al., 2018) metabolism through modulation of regulatory proteins. Previous studies show that miR-20a-5p, which decreased in the TBI group (Table 2), is a negative regulator of APP (Hebert et al., 2009). In addition, a recent study showed that miR-20b-5p targeting of RhoC disturbed AD progression by regulating cell apoptosis, cleaved caspase-3 expression, and cell viability, suggesting that miR-20b-5p might be a curative target for AD (Tian et al., 2021). Further, a recent study showed that a decrease in serum miR-331-3p is correlated with the MMSE scores and proinflammatory cytokine levels in AD patients (Liu and Lei, 2021). The study also showed that miR-331-3p can regulate cell viability and the expression of pro-inflammatory cytokines in Abeta_40_ treated SH-SY5Y cells, supporting a potential neuroprotective role of miR-331-3p. In addition, Burgos et al. considered the correlation between serum and CSF miRNA levels and the extent of plaque and tangle formation in post mortem human brain, i.e. miR-30b-5p, miR-30c-5p, miR-17-5p, miR-374b-5 (Burgos et al., 2014), and additional studies have demonstrated altered levels of miR 30b-5p in post mortem brain (Cogswell et al., 2008; Lau et al., 2013). Together these studies support a potentially mechanistic role for miRNAs in AD pathology, and warrant further investigation into how miRNAs altered in TBI may contribute to AD development.

To understand the signaling networks that may be targeted by the miRNAs identified in each of the group comparisons, we first identified the canonical pathways of the predicted mRNA targets of miRNAs that were significantly different in each group (Table 2). To increase rigor, we used two distinct mRNA target algorithms, TargetScan and miRDB, to identify significant canonical pathways common to both. For the miRNAs differentially expressed in DepTBI vs. ComC (Table 2A) there were 11 overlapping canonical pathways (Figure 5A), 4 of which are relevant to TBI: ephrin receptor, axonal guidance, BMP, and RhoA signaling (Frugier et al., 2012; Patel et al., 2017; Bi et al., 2019; Divolis et al., 2019; Zhao et al., 2019; Greer et al., 2020; Mulherkar and Tolias, 2020; Duman et al., 2021). Likewise, for the miRNAs differentially expressed in DepTBI vs. DepC (Table 2C) there were 20 overlapping canonical pathways (Figure 5C), 8 of which are relevant to TBI: ERK/MAPK, TGF-β, senescence, reelin signaling in neurons, Wnt/β-catenin, eIF4 and p70S6K, PAK, and cyclins and cell cycle regulation (Di Giovanni et al., 2005; Chen et al., 2007; Zhao et al., 2011; Salehi et al., 2018; Divolis et al., 2019; Sen, 2019; Dal Pozzo et al., 2020; Hascup and Hascup, 2020; Sharma et al., 2020; Tan et al., 2020; Schwab et al., 2021). Interestingly, many of these pathways are inter-related. The pro-inflammatory effects of TBI have been shown to be inhibited by activation of TGF-β and BMP (Divolis et al., 2019). Decreases in peripheral levels of Eph4A reduces neuroinflammation and cortical damage of acute TBI (Kowalski et al., 2019). In addition, in postmortem human brain from patients that died after acute closed head injury there is increased Eph4A expression in astrocytes (Frugier et al., 2012), and in Eph4A knockout mice there is a decrease in TBI-induced neurogenesis and amelioration of cognitive impairments compared to wild-type mice (Greer et al., 2020). Furthermore, neuroinflammation and decreases in neurogenesis are two hallmarks of cellular senescence, which was another pathway identified in DepTBI. Furthermore, senescence is a well-established contributing factor to cognitive decline and neurodegeneration in both TBI and AD (Arun et al., 2020; Hascup and Hascup, 2020; Martinez-Cue and Rueda, 2020). Together, these data demonstrate that the predicted mRNA targets for the miRNAs that were present at different levels in DepTBI are relevant to mTBI. The data also suggest that extracellular miRNAs may play a mechanistic role in the neuropathological processes that underlie TBI.

In conclusion, these studies measured changes in the levels of miRNAs in CSF from community and deployed veteran controls with no lifetime history of TBI and veterans who had experienced one or more blast mTBI during the course of deployment in the Iraq/Afghanistan wars. We used complementary measures to analyze the data. Statistical measures revealed changes in CSF miRNA levels in individuals who had experienced one or more mTBI during the course of deployment, as well as changes in CSF miRNAs in the deployed veterans without a history of TBI, suggesting that environmental factors associated with deployment in a war zone may have a durable influence on CSF composition. We then used information theory to examine the correlation between miRNA levels and participant characteristics, and identify potential confounding factors that could influence miRNA measurements in this study. This approach identified several miRNAs that have high information scores with known AD-relevant CSF protein biomarkers (Abeta_42_, tTau, and pTau_181_), age, and smoking, suggesting that these are relevant phenotypic information that should be collected and considered in future studies. It is notable that only some of the relationships identified in the information theory analysis were identified in the statistical analysis, supporting that each approach brings new information regarding miRNAs in TBI and their potential interdependence with phenotypic characteristics. We then employed target prediction and pathway analysis to identify potential targets of the miRNAs altered in each group comparison, and found that a majority of the significant canonical pathways are neurological and may contribute to the effects of TBI. Together, these approaches provide complementary information to support that both post-acute TBI and deployment elicit changes in CSF miRNAs, and that miRNA studies present a means to identify early responses to TBI and track changes that may lead to AD.

## Resource Identification Initiative

**Table:** 

**miRBase**	**SCR_003152**
OHSU Gene Profiling Shared Resource Core Laboratory	SCR_009975
Veriti^TM^ 96-Well Thermal Cycler	SCR_021097
QuantStudio^TM^12K Flex Real-Time PCR System	SCR_021098
QuantStudio^TM^ 12K Flex Real-Time PCR Software	SCR_021096
ExpressionSuite Software	SCR_021095
R packages survival	SCR_003054
Survminer Software	SCR_021094
NormqPCR	SCR_003388
Ingenuity Pathway Analysis	SCR_008653
TargetScan v.7.2	SCR_010845
miRDB	SCR_010848

## Data Availability Statement

The original contributions presented in the study are included in the article/Supplementary Material, further inquiries can be directed to the corresponding author.

## Ethics Statement

The studies involving human participants were reviewed and approved by The Veterans Affairs (VA) Puget Sound Health Care System and the University of Washington Institutional Review Boards. The patients/participants provided their written informed consent to participate in this study.

## Author Contributions

TL, JS, JQ, JL, MR, and EP contributed to conception and design of the study. EP and GL contributed to sample and data sharing. US and JS performed the experiments. TL, NS, SB, JW, and DG performed the analyses. JS and TL wrote the first draft of the manuscript. TL, US, NS, SB, DG, and JS wrote sections of the manuscript. TL, NS, DG, and JS did major editing of the manuscript. All authors contributed to manuscript revisions and read and approved the submitted version.

## Conflict of Interest

The authors declare that the research was conducted in the absence of any commercial or financial relationships that could be construed as a potential conflict of interest.

## Publisher’s Note

All claims expressed in this article are solely those of the authors and do not necessarily represent those of their affiliated organizations, or those of the publisher, the editors and the reviewers. Any product that may be evaluated in this article, or claim that may be made by its manufacturer, is not guaranteed or endorsed by the publisher.

## Footnotes

1
https://CRAN.R-project.org/package=survival

2
www.qiagenbioinformatics.com/products/ingenuity-pathway-analysis
