# Supplementary figures and images for: Cerebrospinal Fluid MicroRNA Changes in Cognitively Normal Veterans With a History of Deployment-Associated Mild Traumatic Brain Injury
Source: Front Neurosci. 2021 Sep 9;15:720778. doi: 10.3389/fnins.2021.720778 (PMC8463659; doi:10.3389/fnins.2021.720778)

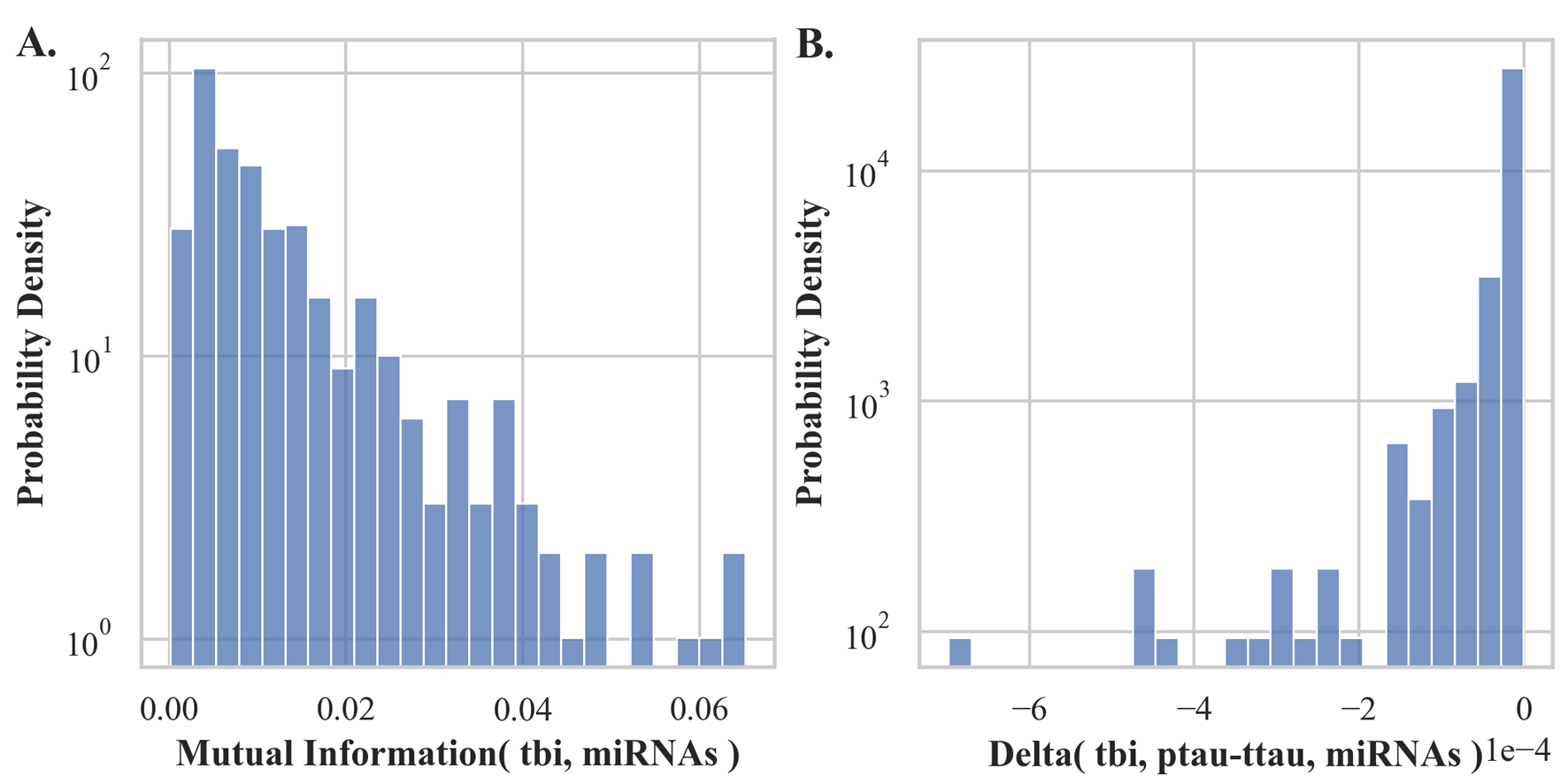

Supplement: Supplementary file 1 [file Image_1.TIFF]
